# Strong association of *TLR2* and *TLR3* polymorphisms with keratoacanthoma and common warts: a case-control study

**DOI:** 10.3325/cmj.2024.65.232

**Published:** 2024-06

**Authors:** Silvana Karabatić Knezović, Dora Knezović, Antonela Matana, Neira Puizina Ivić, Irena Drmić Hofman

**Affiliations:** 1 Secondary Medical School Split, Split, Croatia; 2 Department of Immunology and Genetics, University of Split School of Medicine, Split, Croatia; 3 Department of Health Studies, University of Split, Split, Croatia; 4 Department of Dermatovenerology, University Hospital Split, Split, Croatia; 5 Department of Medical Chemistry and Biochemistry, University of Split School of Medicine, Split, Croatia

## Abstract

**Aim:**

To determine variations in allele and genotype frequencies between keratoacanthoma (KA) and common warts (CW), compared with the control group, in three single nucleotide polymorphisms (SNPs) within the *TLR2*, *TLR3*, and *TLR9* genes.

**Methods:**

This case-control study involved samples from 161 patients with KA, 152 patients with CW, and 469 controls. DNA was isolated from formalin-fixed paraffin-embedded tissue sections. Three SNPs – rs4696480 in *TLR2*, rs7657186 in *TLR3*, and rs35213 in *TLR9* – were genotyped with TaqMan Genotyping Assays on the 7500 Real-Time PCR System.

**Results:**

*TLR2* rs4696480 and *TLR3* rs7657186 were significantly overrepresented in KA and CW compared with controls (*P* < 0.001). The association was stronger for CW than for KA, as evidenced by higher frequencies of the A allele and AA genotype for rs4696480. Both KA and CW patients had higher frequencies of the G allele and GG genotype for rs7657186 than controls. rs7657186 was moderately associated with KA and CW, with the G allele and GG genotype being more prevalent in CW cases, where no AA homozygotes were found.

**Conclusion:**

Genetic variants in *TLR2* (rs4696480) and *TLR3* (rs7657186) genes may affect KA and CW development, influencing immune responses and susceptibility to these skin lesions. Further research is required to elucidate *TLR* expression patterns and their role in KA development.

Keratoacanthoma (KA) is a common benign skin growth, characterized by keratinization, primarily affecting sun-exposed areas. While the exact cause of KA remains unknown, it has been associated with exposure to ultraviolet (UV) radiation, human papillomavirus (HPV) infection, immunosuppression, and genetic factors (1,2). Even though it is histologically similar to squamous cell carcinoma (3), its characteristics such as benign presentation, tendency for spontaneous regression, and the potential etiopathogenic role of HPV (1,4), make it similar to common warts.

Common warts (CW), or *verrucae vulgares*, are benign skin growths caused by HPV infection, typically involving HPV types 1, 2, 4, 27, and 57 (5,6). Similar to KA, they frequently spontaneously regress (7). Immunologically, the host's defense mechanisms recognize viral elements, through pattern recognition receptors like toll-like receptors (TLRs), which triggers an inflammatory response (8). Additionally, genetic variations in TLRs, notably TLR2, TLR3, and TLR9, can detect specific elements of the HPV virus, such as viral proteins or nucleic acids (9). This detection initiates subsequent signaling pathways, resulting in the activation of immune responses (10). Furthermore, polymorphisms in genes such as *TLR* can affect the susceptibility to HPV infection and the development of skin lesions (11,12).

TLRs are expressed by keratinocytes in the epidermis, constantly present or mobile monocytes and macrophages, T and B lymphocytes, mast cells in the dermis, endothelial cells of skin blood vessels, as well as fibroblasts and adipocytes, all contributing to the immune response activation against various microorganisms (13). Multiple studies have noted aberrant *TLR* expression across diverse skin neoplasms, encompassing both inflammatory and neoplastic conditions (14-18). Even though the *TLR* expression profile may vary, the expression of *TLR*1, *TLR2*, *TLR3*, *TLR*4, and *TLR9* is altered in skin tumors (19).

*TLR* variants can impact genes' function, affecting susceptibility to infections and inflammation (20,21). Several *TLR* polymorphisms are also linked to viral infections and UV-induced inflammation in the skin. The *TLR* gene polymorphisms (*TLR2*, *TLR3*, *TLR9*) have been implicated in infectious and inflammatory diseases, and several skin conditions, including psoriasis, atopic dermatitis, systemic lupus erythematosus, diabetic foot ulcers, and skin cancers (22-25). However, there is lack of evidence on their correlation with KA (a lesion of unknown etiology) and CW (a lesion with clearly proven viral etiology).

This study aimed to investigate differences in allele and genotype frequencies between KA and CW, compared with controls in three SNPs within *TLR2*, *TLR3,* and *TLR9* genes (rs4696480, rs7657186, and rs352139, respectively), which may contribute to the immune response to viral pathogens.

## Material and methods

### Material

The research included formalin-fixed paraffin-embedded tissue specimens from 161 KA and 152 CW, obtained after surgical excision, with all samples independently confirmed pathologically by both a pathologist and a dermatopathologist. The tissue specimens were obtained from the Department of Dermatology and Venerology and the Department of Pathology, Forensic Medicine, and Cytology at the University Hospital of Split. Additionally, 469 controls from a larger population-based cohort of 10 001 Dalmatians from the Croatian biobank, who had been previously genotyped, were selected and matched on age and sex with the KA and CW groups. All procedures were approved by the Ethics Committee of the University Hospital of Split.

### DNA isolation from FFPE tissue

In brief, DNA samples were extracted from four to five 4-μm sections of formalin-fixed paraffin-embedded (FFPE) slides, following the NucleoSpin Tissue Kit (Macherey-Nagel, Dueren, Germany) manufacturer’s protocol. The concentration and purity of DNA were assessed by using a NanoDrop 1000 spectrophotometer (ThermoFisher Scientific, Wilmington, DE, USA), followed by single nucleotide polymorphism (SNP) genotyping.

### SNP genotyping

Three SNPs, rs4696480, rs7657186, and rs352139 in the *TLR2*, *TLR3*, and *TLR9* genes, respectively, were genotyped with real-time polymerase chain reaction (RT-PCR) by using the TaqMan Genotyping Master Mix and TaqMan SNP Genotyping Assays (ThermoFisher Scientific Wilmington, DE, USA) (Table 1) on the 7500 Real-time PCR System (Applied Biosystems, Foster City, CA, USA), following the manufacturer's instructions.

**Table 1:** Detection of single nucleotide polymorphisms (SNPs) of *TLR2*, *TLR3* and *TLR9* genes

Gene	SNP ID	Chromosome location (GRCh38)	TaqMan Assay ID	Nucleotide change
*TLR2*	rs4696480	Chr.4: 153685974 - 153685974	C__27994607_10	*T*> A
*TLR3*	rs7657186	Chr.4: 186072885 - 186072885	C__27310255_20	G>A
*TLR9*	rs352139	Chr.3: 52224356 - 52224356	C___2301953_10	*T*> C

### Statistical analysis

The differences between the groups in epidemiological data (age and sex) were assessed with a χ^2^ test. Allele and genotype frequencies were calculated by direct counting. Allele frequencies and the Hardy-Weinberg equilibrium (HWE) were calculated separately for each population by using VCFtools (26). The differences in allele and genotype SNP frequencies, as well as the association with sex, were assessed using the χ^2^ and Fisher exact *P* value tests with PowerMarker V.3.25. software (27). ANOVA was implemented to estimate the relationship between age and genotypes according to observed skin lesions. Logistic regression was used to estimate the correlation between age and skin lesions. *P* values <0.05 were considered statistically significant. The analysis was performed with the MedCalc statistical software V. 22 (MedCalc Software Ltd, Ostend, Belgium).

## Results

The characteristics of patients with KA and CW are shown in Table 2. KA and CW were equally distributed between sexes (*P* = 0.423). KAs were more prevalent among patients over 76 years, while CWs were more prevalent among patients under 46 years (*P* < 0.001).

**Table 2:** Age and sex of patients with keratoacanthoma (KA) and common warts (CW)

Variables		KA (n = 161)	CW (n = 152)
Age, years (n)	<46	19	60
	46-65	41	41
	66-76	49	32
	>76	52	18
Sex (n)	female	69	72
	male	92	80

The frequencies of alleles and genotypes observed in the KA, CW, and control groups are shown in Table 3. The genotypes of three SNPs in all sample groups followed the HWE.

**Table 3:** Allele and genotype frequencies in patients with keratoacanthoma (KA) and common warts (CW), and controls*

Gene/SNP	Genotype/allele	KA		CW		Controls	
		n	f	n	f	n	f
***TLR2*** **rs4696480**	AA	88	0.568	91	0.628	114	0.234
	AT	30	0.194	21	0.115	245	0.522
	TT	37	0.239	33	0.228	110	0.235
	A	206	0.665	203	0.7	473	0.504
	T	104	0.335	87	0.3	465	0.498
***TLR3*** **rs7657186**	GG	117	0.778	120	0.857	297	0.633
	GA	30	0.197	20	0.143	150	0.32
	AA	5	0.033	0	0	22	0.047
	G	264	0.868	260	0.929	744	0.793
	A	40	0.132	200	0.071	194	0.207
***TLR9*** **rs352139**	CC	49	0.303	60	0.395	172	0.367
	CT	90	0.563	69	0.454	229	0.488
	TT	21	0.131	23	0.151	68	0.145
	C	188	0.588	189	0.622	573	0.611
	T	132	0.413	115	0.378	365	0.389

*Abbreviations: SNP – single nucleotide polymorphism; n – number of genotypes and alleles; f – frequency of genotypes and alleles.

The allele and genotype frequencies of *TLR2* rs4696480 and *TLR9* rs352139 did not significantly differ between KA and CW skin lesions, while the frequencies of *TLR3* rs7657186 were higher in the CW group (Table 4). This can be attributed to significantly higher relative frequencies of the G allele (0.868 vs 0.929) and GG genotype (0.778 vs 0.857) on rs7657186 in the CW group. Furthermore, in patients with CW, there were no AA homozygotes.

**Table 4:** Differences in allele and genotype frequencies of the observed single nucleotide polymorphisms (SNPs) between patients with keratoacanthoma (KA) and common warts (CW)

Lesion	SNP	Allele value*	Allele P*	Genotype value*	Genotype P*	Allele P^†^	Genotype P^†^
KA/CW	rs4696480	0.77	0.378	1.44	0.487	0.332	0.487
KA/CW	rs7657186	5.72	0.017	6.56	0.038	0.012	0.033
KA/CW	rs352139	0.66	0.418	3.52	0.172	0.443	0.181

*χ^2^ test.

**†**Fisher exact test.

rs4696480 and rs7657186 at allelic and genotype levels were strongly associated with both KA and CW (Table 5). The associated SNPs were significantly overrepresented in KA and CW compared with the control group. The CW group was more strongly associated with rs7657186 compared with KA. Patients with KA showed significantly higher frequencies of the A allele (0.665 vs 0.504) and AA genotype for rs4696480 (0.568 vs 0.234) than the control group. When it comes to rs7657186, the KA group had significantly higher frequencies of the G allele (0.868 vs 0.793) and GG genotype (0.778 vs 0.633) than the control group.

**Table 5:** Differences in allele and genotype frequencies of the observed single nucleotide polymorphisms (SNPs) in patients with keratoacanthoma (KA) and common warts (CW) compared with controls

Lesion	SNP	Allele value*	Allele P*	Genotype value*	Genotype P*	Allele P^†^	Genotype P^†^
KA	rs4696480	24.90	<0.001	67.63	<0.001	<0.001	<0.001
KA	rs7657186	8.50	0.003	9.67	0.008	0.002	0.006
KA	rs352139	0.43	0.510	2.44	0.296	0.510	0.275
CW	rs4696480	34.30	<0.001	85.51	<0.001	<0.001	<0.001
CW	rs7657186	27.29	<0.001	26.56	<0.001	<0.001	<0.001
CW	rs352139	0.11	0.736	0.55	0.758	0.733	0.736

*χ^2^ test.

†Fisher exact test.

Patients with CW showed significantly higher frequencies of the A allele (0.7 vs 0.504) and AA genotype for rs4696480 (0.628 vs 0.234) than the control group. Regarding rs7657186, the CW group had significantly higher frequencies of the G allele (0.929 vs 0.793) and GG genotype (0.857 vs 0.633) than the control group.

No association was found between the observed SNPs and skin lesions based on sex (Table 6). No significant correlations were noticed between age and genotypes according to the observed skin conditions. After applying logistic regression analyses to estimate the association between age and skin lesions, we found a significant regression coefficient (null model -2 log likelihood = 404.305; full model -2 log likelihood = 350.441; χ^2^ = 53.864, *P* < 0.0001, R^2^ = 0.204). On average, patients with KA were older (68 years) than patients with CW (51 years).

**Table 6:** Differences in allele and genotype frequencies of the observed single nucleotide polymorphisms (SNPs) in patients with keratoacanthoma (KA) and common warts (CW) according to sex

Lesion	SNP^†^	Allele value*	Allele P*	Genotype value*	Genotype P*	Allele P^†^	Genotype P^†^
KA	rs4696480	0.24	0.627	0.20	0.906	0.629	0.900
KA	rs7657186	0.16	0.691	0.71	0.699	0.749	0.792
KA	rs352139	0.13	0.719	5.47	0.065	0.631	0.075
CW	rs4696480	2.70	0.101	2.18	0.336	0.088	0.351
CW	rs7657186	0.02	0.894	0.02	0.890	1.000	1.000
CW	rs352139	0.36	0.549	0.71	0.703	0.588	0.762

*χ^2^ test.

†Fisher exact test.

## Discussion

Our study revealed a strong association of rs4696480 and rs7657186 with both skin conditions, compared with controls, with CW exhibiting a stronger link with rs7657186 than KA.

*TLR*s are essential components of the innate immune system, recognizing pathogen-associated molecular patterns and initiating immune responses against them (8). In the case of CW, primarily caused by HPV, excessive or dysregulated inflammation can contribute to wart formation, with *TLR*s being potentially involved in regulating this inflammatory equilibrium (28). A systematic review by Neagu et al summarized scientific data on the HPV's role in keratinocyte skin cancers. Among 321 patients with KA, 37 tested positive for HPV (11.52%), mostly beta type (29). HPV was also associated with specific cases of KA and cutaneous squamous cell carcinoma (30). Overall, *TLR*s play important roles in recognizing HPV viruses and initiating immune responses, which highlights their importance in the host antiviral defense (28).

*TLR* gene polymorphisms rs4696480, rs7657186, and rs352139 have been implicated in various diseases, including skin diseases (20-25). Given that KA commonly appears on photoexposed skin (1), polymorphisms in *TLR3* (rs7657186) (31) and *TLR9* (rs352139) (32), expressed in photoexposed skin, as well as *TLR2* polymorphism (rs4696480) associated with specific skin conditions (23,33,34), could reflect differential response to the interplay between viral exposure and UV induced skin damage (35,36).

In this study, the CW group had a higher prevalence of G allele on *TLR3* rs7657186, and there were no individuals with AA homozygosity in this group. These findings align with recent research indicating that when *TLR3* is stimulated by external or internal triggers, skin and immune cells play a significant role in the development of infectious or inflammatory skin conditions, such as viral infections, allergies, and dermatitis (37). *TLR3* is involved in host anti-viral immunity, functioning defensively and offensively by recognizing double-stranded RNA, a common replication intermediate among numerous viruses (38). The rs352139 variant in *TLR3* may impact its ability to detect viral pathogens or modulate immune signaling pathways in response to viral infections in photoexposed skin (39).

This case-control study found an evident association between *TLR2* rs4696480 and *TLR3* rs7657186 for both KA and CW compared with controls at both allelic and genotype levels. Specifically, individuals with KA had notably higher frequencies of the A allele and AA genotype for rs4696480 than controls, while those with CW exhibited similar patterns. For rs7657186, patients with CW demonstrated markedly higher frequencies of the G allele and GG genotype compared with those with KA, which suggests a stronger correlation with this SNP.

While *TLR3* has been initially investigated in the context of viral infections, its ability to recognize endogenous ligands released during tissue damage and inflammation could suggest a role in the pathogenesis of KA (40,41).

Similarly, *TLR2* has been associated with responding to viral ligands and susceptibility to various infectious and inflammatory conditions (42). In terms of skin diseases, *TLR2* polymorphisms, such as rs4696480, have been connected with altered immune responses and increased susceptibility to acne vulgaris (19). The same genetic variant has also been studied in relation to psoriasis, a chronic inflammatory skin condition (23).

Even though *TLR9* is responsible for recognizing viral DNA (42), there were no significant differences between the examined groups in rs352139 polymorphism, whether compared with each other or the control group.

While the specific roles of *TLR*s in KA pathogenesis remain to be fully elucidated, dysregulated TLR signaling may contribute to the inflammatory microenvironment observed in KA lesions. The results may vary with a broader analysis of *TLR* polymorphisms and a larger cohort of individuals with KA and CW. Further research is needed to clarify TLR expression patterns and their functional significance in KA.

In conclusion, certain genetic variants within *TLR2* and *TLR3* genes may contribute to the etiology of KA and CW, potentially affecting immune responses and predisposition to these dermatological conditions.
